# Soil nutrient content dominates short-term vegetation changes in alpine tundra of Changbai Mountains

**DOI:** 10.3389/fmicb.2024.1422529

**Published:** 2024-08-16

**Authors:** Shanfeng Xing, Wen J. Wang, Lei Wang, Haibo Du, Zhengfang Wu, Shengwei Zong, Yu Cong, Shengjie Ba

**Affiliations:** ^1^State Key Laboratory of Black Soils Conservation and Utilization, Northeast Institute of Geography and Agroecology, Chinese Academy of Sciences, Changchun, China; ^2^Key Laboratory of Geographical Processes and Ecological Security in Changbai Mountains, Ministry of Education, School of Geographical Sciences, Northeast Normal University, Changchun, China

**Keywords:** short-term vegetation change, soil nutrient content, herb encroachment, alpine tundra, Changbai Mountains

## Abstract

Alpine tundra, covering 3% of the Earth’s land surface, harbors approximately 4% of higher plant species. Changes in this vegetation significantly impact biodiversity and ecosystem services. Recent studies have primarily focused on large-scale and long-term vegetation changes in polar and high-latitude regions. However, the study of short-term vegetation changes and their primary drivers has received insufficient attention in alpine tundra. This study aimed to investigate vegetation changes and their dominant drivers in the alpine tundra of Changbai Mountains-located at the southern edge of the alpine tundra distribution in Eastern Eurasia-over a short period by re-surveying permanent plots in 2019 and comparing them with data from 2014. The results showed that significant changes were observed in alpine tundra vegetation during the study period. The importance values of typical alpine tundra plants such as *Rhododendron chrysanthum*, *Vaccinium uliginosum*, and *Dryas octopetala* decreased noticeably, while those of herbaceous species such as *Deyeuxia angustifolia* and *Sanguisorba sitchensis* increased significantly. Species richness, diversity, and evenness at different altitudinal gradients showed varying degrees of increase. A distinct expansion trend of herbaceous species was observed in the alpine tundra, contributing to a shift in plant community composition toward herbaceous dominance. This shift might result in the meadowization of the dwarf shrub tundra. Our findings further revealed that soil nutrients rather than climate factors, dominated the changes of plant communities over a short period. These findings provide scientific references for the conservation and management of biodiversity, as well as for projecting future vegetation dynamics in alpine tundra.

## 1 Introduction

Alpine tundra, an integral part of alpine ecosystems, covers 3% of the Earth’s land surface and harbors approximately 4% of higher plant species (Körner, 1999). Alpine ecosystems are highly sensitive to climate change because the plant species within these ecosystems are adapted to harsh climate conditions, such as low temperatures and high humidity (Walker et al., 2006; Danby et al., 2011; Alatalo et al., 2017; Czortek et al., 2018; Rantanen et al., 2022). Numerous studies have documented notable shifts in vegetation composition, biomass, and diversity in tundra regions for the past decades, driven by climate change (Lenoir and Svenning, 2014), environmental change (Chu et al., 2016; López-Angulo et al., 2019), and human activities such as land use change (Steinbauer et al., 2018), grazing (Dunwiddie and Research, 1977), and tourism (Walther et al., 2005). These changes could alter biophysical and biogeochemical processes, affecting the exchanges of energy, water, carbon, and nutrients between the soil and the atmosphere (Tarnocai et al., 2009; Myers-Smith et al., 2011). Therefore, understanding the changes in alpine tundra vegetation and their underlying mechanisms is essential for accurately forecasting, alleviating, and adapting to the influences of future environmental shifts on these ecosystems (Rixen et al., 2022).

Recent studies on vegetation changes in alpine tundra have predominantly focused on polar and high-latitude regions (Hallinger et al., 2010; Salminen et al., 2023; Lagergren et al., 2024). Low-elevation vegetation in polar regions and high latitudes was encroaching upon alpine tundra (Sturm et al., 2001; Tape et al., 2006; Formica et al., 2014; Shevtsova et al., 2020), which gradually reduced the area of alpine tundra. Shevtsova et al. (2020) observed vigorous shrub expansion in the northeastern Siberian tundra. Meanwhile, studies focusing on high-mountain vegetation change typically employed large-scale research methods, facilitating the identification and monitoring of spatial and temporal patterns in vegetation changes (Ju and Masek, 2016; Nill et al., 2022). For example, Nill et al. (2022) analyzed Landsat data from 1984 to 2020 and found that shrub cover increased on average by 1.4–4.2% per decade across the entire ecological zone of western Canada. However, these large-scale methods failed to capture subtle vegetation changes and processes, as they were unable to detect changes in the composition, structure, and species diversity within plant communities (Myers-Smith et al., 2019). Establishing permanent monitoring plots could help overcome the limitations associated with large-scale studies by providing a more detailed and accurate representation of actual ecosystem dynamics (Kapfer et al., 2016). By consistently and regularly monitoring these plots, researchers could quantitatively evaluate shifts in the composition and diversity of plant communities (Pauli et al., 2012; Gazol et al., 2017). This approach was particularly effective when permanent plots were established along elevation gradients, as it allowed for a more precise analysis of the dynamics across different vegetation zones (Metcalfe et al., 2018; Walker et al., 2018). Given the high spatial heterogeneity and temporal variability of tundra landscapes (Epstein et al., 2012; Reichle et al., 2018), employing this method is essential to thoroughly investigate changes in the composition, structure, and species diversity of alpine tundra.

Climate warming has long been regarded as the primary driver of vegetation changes (Pickering et al., 2008; Grabherr et al., 2010; Danby et al., 2011; Lenoir and Svenning, 2014). For example, Pickering et al. (2008) demonstrated that climate warming led to shifts in the distribution of thermophilic shrubs, herbs, and invasive weeds towards higher elevations. However, these studies typically analyzed the drivers of long-term vegetation changes (Holzinger et al., 2007), and the impacts of long-term climate change may interact with other environmental changes (Hamid et al., 2020), such as disturbances from typhoons, land use changes, and soil nutrient variations. To avoid biased conclusions that might arise from confounding factors, we focused on investigating the driving factors of plant community changes at short time scales. Short-term studies of changes in plant communities often relied on controlled experiments, such as warming experiments (Klein et al., 2004) and nitrogen fertilization (Britton and Fisher, 2008). However, this approach could only evaluate the impact of controlled environmental factors on plant communities and fails to identify the dominant factors that actually drive changes in these communities (Clark et al., 2007). There is a knowledge gap regarding actual vegetation changes and their dominant drivers over a short period in alpine dwarf-shrub tundra.

The alpine tundra of Changbai Mountains (ATCBM), located at the southern edge of the alpine tundra distribution in Eastern Eurasia, represents one of the most archetypal mountain tundra vegetation ecosystems in China (Huang and Li, 1984). The mean growing season temperature showed a significant increasing trend for the past six decades, with a rate of 0.23°C/decade in the ATCBM, which was higher than the average global surface warming rate over the past 50 years (Wang et al., 2019). Vegetation changes in the ATCBM have increasingly attracted attention in recent years, such as the upward migration of tree line (Du et al., 2017) and the herb encroachment (Jin et al., 2019a). Wang et al. (2019) evaluated changes in the vegetation of the ATCBM for the past three decades by comparing the historical and current vegetation survey results. However, this comparison using non-permanent plot surveys introduced considerable uncertainty into the observed vegetation changes and hindered the attribution analysis of these changes (Kapfer et al., 2016). Therefore, conducting regular field permanent plot surveys is crucial for monitoring changes in alpine tundra vegetation and associated environmental factors, thereby identifying the dominant drivers of vegetation change.

In this study, we established permanent plots in the ATCBM and conducted detailed monitoring in 2014 and 2019. By comparing the results from these two surveys, we systematically analyzed changes in the tundra vegetation over five years and evaluated the underlying mechanisms driving these changes within a short period. We proposed the following hypotheses: (1) the vegetation underwent significant changes over a short period in the ATCBM, characterized by the rising importance of herbaceous plants and increased species diversity; (2) the encroachment of herbaceous species significantly altered the composition and structure of the native plant communities; (3) changes in soil nutrient content, rather than climate factors, were the more significant drivers of alpine vegetation changes over a short period.

## 2 Materials and methods

### 2.1 Study area

**FIGURE 1:**
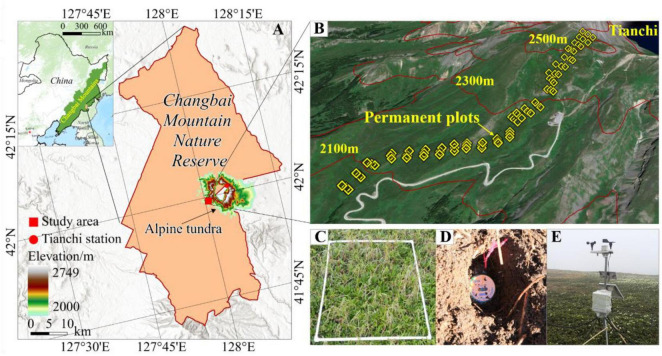
Location of the study area in the ATCBM **(A)** and the setup of plots **(B,C)**, automatic weather stations **(D)**, and temperature recorder (Tidbit@Tv2) **(E)**.

The alpine tundra is situated on the main peak of Changbai Mountains, at an altitude exceeding 2000 meters (m), spanning from 41° 56’N to 42°04’N and 127°58’E to 128°11’E (Figure 1A). This region experiences a harsh alpine tundra climate, characterized by low temperatures (5.8°C) and abundant precipitation (958 mm) during the growing season (June to September), coupled with a winter that extends over eight months (Hao et al., 2001). The average snow depth reaches about 1 m during winter. The soil in the tundra is notably shallow, typically not exceeding 30 cm in depth, and characterized as coarsely bony, thinly layered, and poorly stratified (Huang and Li, 1984). Vegetation in the area is sparse, primarily composed of a few dwarf shrubs, mosses, and lichens. Prominent plant species include dwarf shrubs such as *Rhododendron chrysanthum*, *Vaccinium uliginosum*, and *Dryas octopetala*, along with herbaceous plants like *Carex pachyneura*, *Sanguisorba sitchensis*, and *Saussurea tomentosa* (Huang and Li, 1984). Recent decades of rapid warming have prompted shifts in alpine tundra vegetation, particularly the upward shift of the tree line (Du et al., 2017) and the increasing prevalence of herbaceous plant encroachment (Wang et al., 2019).

### 2.2 Vegetation and soil sampling

The field survey utilized a systematic sampling method, establishing permanent quadrats along a transect. At each altitude range within this transect, three replicate 1m x 1m plots were set up, spaced 25 m apart (Figure 1C). These plots were positioned at 100-meter intervals along the slope, extending from the tree line at 2050 m to the upper edge of the tundra at 2560 m. Each set contains 25 quadrats, resulting in a total of 75 quadrats (Figure 1B). To minimize human disturbance and accurately capture changes in plant communities, each set was located at least 50 m away from the nearest highway. The establishment of this transect and permanent quadrats, as well as the initial detailed vegetation survey, occurred in 2014 and 2015 (Wang et al., 2019). We conducted a re-survey of these permanent quadrats in 2019. To accurately capture changes in plant species, we used the same indicators as the previous survey of the quadrats, such as plant name, coverage, number of plants, and plant height. We also used soil auger (3 cm diameter) to collect soil samples from each quadrat to analyze the physicochemical properties of soil, such as particle size (clay, silt, and sand), organic matter (Org), total nitrogen (TN), available nitrogen (AN), available phosphorus (AP), available potassium (AK), and carbon-to-nitrogen ratio (C/N). Specifically, particle size (clay, silt, and sand) was analyzed using the Bouyoucos hydrometer method (Pauwels et al., 1992); Org was determined by using titrimetric methods, and its contents was estimated from the organic carbon content by multiplying by a factor of 1.724 (Walkley and Black, 1934); TN was evaluated using the Kjeldahl distillation method, while AN was assessed through titration (Bremner and Mulvaney, 1982); AP was determined using the Olsen method (Lajtha and Jarrell, 1999); AK was measured using a flame photometer (Olorunfemi et al., 2016). Moreover, the soil microbial community was characterized using Phospholipid Fatty Acid analysis (Bligh and Dyer, 1959; Frostegård et al., 1991).

### 2.3 Climate data

Since the mid-1980s, herbaceous plants such as *D. angustifolia* have begun encroaching upon the alpine dwarf-shrub tundra from lower altitudes (Zong et al., 2016). Consequently, we utilized historical climate data spanning from 1985 to 2020, acquired from Tianchi Station (42°01’N, 128°05’E), located about 4 kilometers from our research site at an elevation of 2623 m. It should be noted that winter climate data from this station have been unavailable since 1989. These data were subjected to quality control and homogeneity assessments by the National Meteorological Information Center prior to their release.

To accurately monitor soil temperature, we buried a temperature recorder (Tidbit@Tv2) at a depth of 5 cm within each quadrat starting in 2015 (Figure 1E). The soil temperature data used in this study cover five complete growing seasons from June to September, spanning from 2015 to 2020. Additionally, we positioned two automatic weather stations (WeatherHawk 610, Campbell Scientific, Logan, UT, USA) at altitudes of 2135 m and 2246 m to monitor precipitation in the tundra (Figure 1D). Our observations indicated that precipitation increases with altitude in the Changbai Mountains. During the growing seasons from 2014 to 2019, mean precipitation at both elevations were 1270 mm and 1348 mm, respectively. We used the established relationship between precipitation and elevation to interpolate the precipitation value for each plot (Hao et al., 2001; Wang et al., 2019).

### 2.4 Data analysis

In this study, we applied importance value index (IVI) to assess the structural composition and community dynamics within the tundra vegetation of the Changbai Mountains (Lu et al., 2011; Niu et al., 2017). We calculated the IVI for each species using the following formula (Equation 1):


(1) I⁢V⁢I=R⁢d+R⁢h+R⁢c

Relative density (Rd) is calculated by dividing the number of individuals of a particular plant species by the total number of individuals across all species and multiplying the result by 100. Relative height (Rh) is determined by dividing the height of a particular plant species by the cumulative height of all species and then multiplying by 100. Similarly, Relative cover (Rc) is calculated by dividing the cover of a single species by the total cover of all species and then multiplying by 100. By calculating these metrics, we systematically analyze the species’ IVI in descending order to identify their dominance within the vegetation.

To facilitate the analysis of tundra vegetation changes with altitude, we divided the 75 quadrats into five altitudinal gradients: 2050–2150 m, 2150–2250 m, 2250–2350 m, 2350–2450 m, and 2450–2550 m. This division was established to facilitate a comparative analysis of species diversity across each altitudinal gradient. Specifically, we aimed to compare the species richness, diversity and evenness between 2014 and 2019 across each elevation gradient. The calculation formulas for the Patrick index (R), Shannon-Wiener index (H′) and Pielou index (E) of species richness, diversity and evenness were as follows (Equations 2–4; Ma et al., 1995):

Patrick index:


(2) R=S

Shannon-Wiener index:


(3) $$H^{\prime }=-\sum_{i=1}^{s}P_{i}\,\text{ln}\,P_{i}$$

Pielou index:


(4) $$E=H^{\prime }/\text{ln}\,S$$

Where: S is the number of species in the sample plots, and P_*i*_ is the relative IVI of species i. The Shannon-Wiener index and Pielou index represent α-diversity.

In our study, we used ordination analysis methods to examine the connections between vegetation dynamics and environmental factors in the ATCBM. We first employed detrended correspondence analysis (DCA) to determine the gradient length of the ordination axes and found that the maximum value exceeded 4 for the first four axes. Therefore, we utilized canonical correspondence analysis (CCA). This ordination technique, a key statistical method, was conducted using R software (version 3.0.1, R Foundation for Statistical Computing, Vienna, Austria), which was widely recognized for its robust capabilities in ecological data analysis (Greig-Smith, 1983; Šmilauer and Lepš, 2014). The CCA allowed us to directly correlate changes in plant species with variations in environmental parameters, such as Org, TN, AN, AP, AK, C/N, precipitation (PRE), growing season temperature (TEMP), diurnal temperature range (DTR), growing season length (GSL), and winter snow protection (WSP). A paired t-test was conducted to assess the statistical differences in vegetation and environmental changes between the two surveys, using a significance threshold of p < 0.05.

## 3 Results

### 3.1 Changes in tundra vegetation

#### 3.1.1 Changes in the importance value of plant species

The IVI of plant species exhibited significant changes (*p* < 0.05) from 2014 to 2019 (Table 1). Native plants, such as *R. chrysanthum*, *V. uliginosum*, and *D. octopetala*, experienced varying degrees of declines in Rd, Rh, and Rc over the 5-year period in the alpine tundra. These declines resulted in a reduction in their IVI, with rates of decrease at 21.36%, 17.71%, and 13.68%, respectively. Despite experiencing the highest rate of decline in IVI, *R. chrysanthum* still ranked first in species importance, significantly surpassing the other species. While encroaching herbaceous plants such as *Deyeuxia angustifolia* and *S. sitchensis* showed different degrees of increase in their Rd, Rh, and Rc over the 5-year period, these changes resulted in a corresponding rise in their IVI, with rates of increase of 22.14% and 7.46%, respectively. In the past 5 years, among the dominant species (with IVI greater than 2), only 30.8% (*R. chrysanthum, S. sitchensis, D. angustifolia*, and *Geranium dahuricum*) maintained the same ranking of IVI, which indicated that the species composition and structure of the tundra vegetation was not stable but rather undergoing continual change.

**TABLE 1:** Changes in Rd, Rh, Rc, IVI, and ranking of different plant species from 2014 to 2019 in the ATCBM.

Plant species	Rd		Rh		Rc		IVI		Rank change
	2014	2019	2014	2019	2014	2019	2014	2019	
*Rhododendron chrysanthum*	31.76	22.37	6.24	6.12	25.84	21.71	21.28	16.73	0
*Sanguisorba sitchensis*	7.97	8.02	9.65	9.76	10.32	12.25	9.31	10.01	0
*Deyeuxia angustifolia*	7.66	6.54	11.09	10.10	5.28	4.47	7.42	9.06	0
*Sanguisorba teriuifolia*	4.96	7.96	7.49	6.07	6.20	9.12	6.81	5.69	−1
*Ligularia jamesii*	4.37	4.27	6.23	6.36	3.99	3.67	5.14	5.84	1
*Vaccinium uliginosum*	4.43	4.82	1.51	1.11	4.75	6.34	3.62	2.98	−3
*Trollius japonicus*	3.65	2.90	6.50	4.53	5.70	4.93	3.58	2.59	−4
*Saussurea tomentosa*	4.37	3.81	2.31	2.89	1.94	2.10	3.56	3.61	1
*Polygonum viviparum*	2.79	1.90	3.25	3.15	4.57	4.41	3.19	3.02	1
*Dryas octopetala*	2.54	4.13	0.46	0.44	1.69	1.98	2.61	2.25	−2
*Rhodiola cretinii*	1.90	1.34	3.35	2.56	2.35	1.90	2.53	2.89	1
*Carex pachyneura*	1.94	2.37	2.99	4.49	1.78	1.46	2.26	3.85	6
*Geranium dahuricum*	2.65	2.85	2.44	2.29	1.88	1.70	2.05	2.04	0

#### 3.1.2 Changes in species diversity along elevation gradients

**FIGURE 2:**
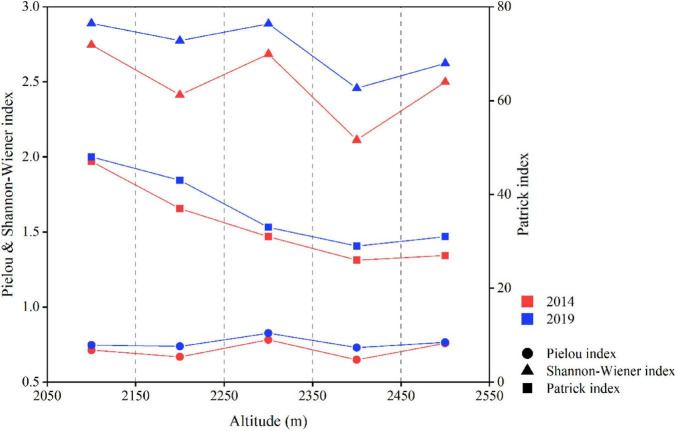
The changes in species richness, diversity, and evenness of vegetation along the altitudinal gradient from 2014 to 2019 in the ATCBM.

The species richness of tundra vegetation exhibited a declining trend with rising altitude on Changbai Mountains (Figure 2). In both 2014 and 2019, species richness in the tundra vegetation on Changbai Mountains peaked at a maximum of 47 and 48, respectively, at an altitude range of 2050–2150 m, while the minimum values recorded were 26 in 2014 and 29 in 2019 at an altitude range of 2350–2450 m. Between 2014 and 2019, species richness increased at each elevation gradient. Specifically, the minimal increase was observed in the 2050–2150 m elevation range, with an addition of just one species, whereas the most substantial increase occurred in the 2150–2250 m range, where species richness expanded by six species. In both 2014 and 2019, the Shannon-Wiener index exhibited a consistent decreasing trend with rising altitude, peaking at 2050–2150 m and reaching its lowest value at 2350–2450 m. From 2014 to 2019, the Shannon-Wiener index increased at varying degrees across different altitudinal gradients, with the most significant increase observed at 2150–2250 m. In both 2014 and 2019, the Pielou index displayed consistent trends with increasing altitude, peaking at 2250–2350 m and reaching a minimum at 2350–2450 m. During this period, the Pielou index exhibited varying degrees of increase across the elevation gradients, with the most significant change observed in the 2350–2450 m range.

#### 3.1.3 Changes in species composition and coverage of typical plant communities

**FIGURE 3:**
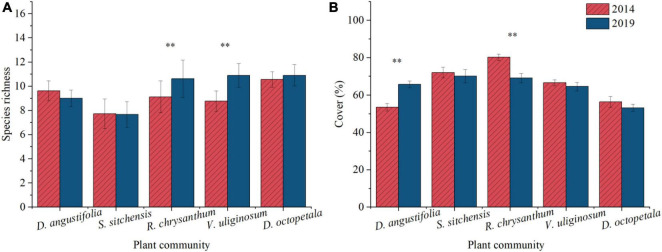
Changes in species abundance **(A)** and dominant species cover **(B)** from 2014 to 2019 in typical plant communities. **represents a difference that is significant at the 0.05 level.

**FIGURE 4:**
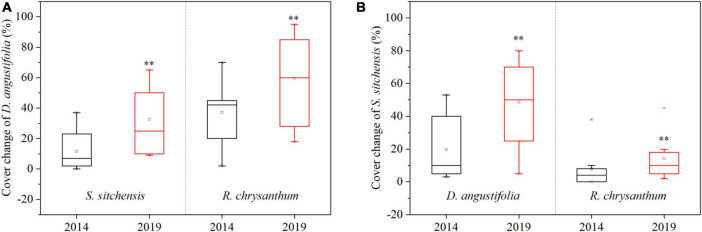
Changes in species coverage of *D. angustifolia* from 2014 to 2019 in the *S. sitchensis* and *R. chrysanthum* communities **(A)**; Changes in species coverage of *S. sitchensis* from 2014 to 2019 in the *D. angustifolia* and *R. chrysanthum* communities **(B)**. **represents a difference that is significant at the 0.05 level.

Five representative plant communities including three native dwarf shrub communities (*S. sitchensis*, *R. chrysanthum*, and *V. uliginosum*) and two herbaceous communities (*D. angustifolia* and *D. octopetala*) were chosen to examine changes in species composition and cover at the community scale over the period from 2014 to 2019. Species richness decreased in the *D. angustifolia* community and remained essentially unchanged in the *S. sitchensis* community. In contrast, it increased in the *R. chrysanthum*, *V. uliginosum*, and *D. octopetala* communities, with significant increases (*p* < 0.05) observed in the *R. chrysanthum* and *V. uliginosum* communities (Figure 3A). The coverage of dominant species significantly increased (*p* < 0.05) in the *D. angustifolia* community, while these exhibited declining trends in the *S. sitchensis*, *R. chrysanthum*, *V. uliginosum*, and *D. octopetala* communities. Notably, the coverage of dominant species showed a significant decline (*p* < 0.05) in the *R. chrysanthum* community (Figure 3B). Simultaneously, we investigated changes in species cover of *D. angustifolia* in the *S. sitchensis* and *R. chrysanthum* communities, as well as changes in species cover of *S. sitchensis* in the *D. angustifolia* and *R. chrysanthum* communities. We found a significant increase (*p* < 0.05) in the coverage of *D. angustifolia* in both the *S. sitchensis* and *R. chrysanthum* communities (Figure 4A), as well as a significant increase (*p* < 0.05) in the coverage of *S. sitchensis* in both the *D. angustifolia* and *R. chrysanthum* communities (Figure 4B).

### 3.2 Changes in environmental factors

#### 3.2.1 Changes in climate factors

**FIGURE 5:**
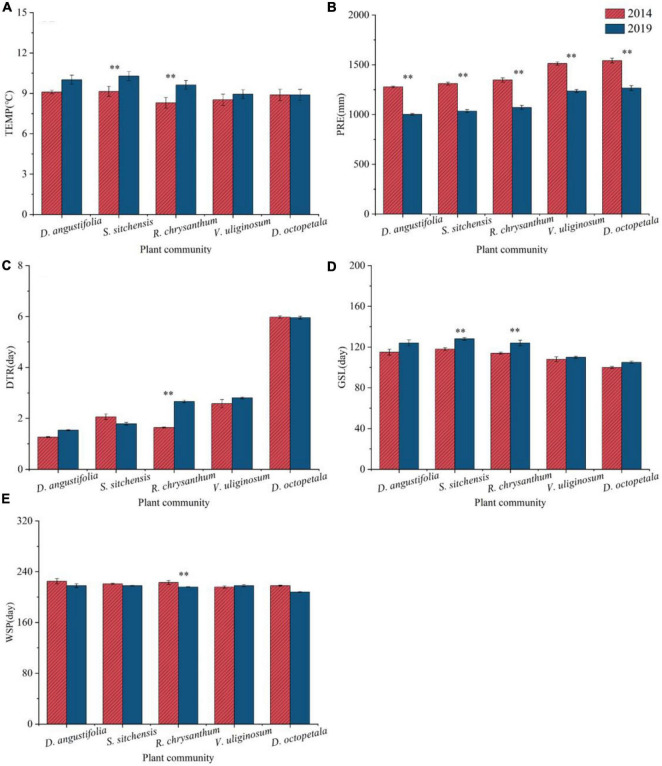
Changes in climate factors [TEMP **(A)**, PRE **(B)**, DTR **(C)**, GSL **(D)**, WSP **(E)**] from 2014 to 2019 in different plant communities in the ATCBM. **represents a difference that is significant at the 0.05 level.

From 2014 to 2019, various climate factors including TEMP, PRE, DTR, GSL, and WSP showed different variations across five plant communities (Figure 5). TEMP increased across all communities, with significant increases in the *S. sitchensis* and *R. chrysanthum* communities. PRE decreased significantly in all plant communities (*p* < 0.05). DTR increased in the *D. angustifolia*, *R. chrysanthum*, and *V. uliginosum* communities, with a significant increase in the *R. chrysanthum* community (*p* < 0.05); however, it decreased in the *S. sitchensis* and *D. octopetala* communities. In line with the TEMP trend, GSL also increased in all communities, particularly with significant increases in the *S. sitchensis* and *R. chrysanthum* communities. WSP decreased in four of plant communities, except for an increase in the *V. uliginosum* community; a significant decrease was noted in the *R. chrysanthum* community (*p* < 0.05).

#### 3.2.2 Changes in soil nutrient content

**FIGURE 6:**
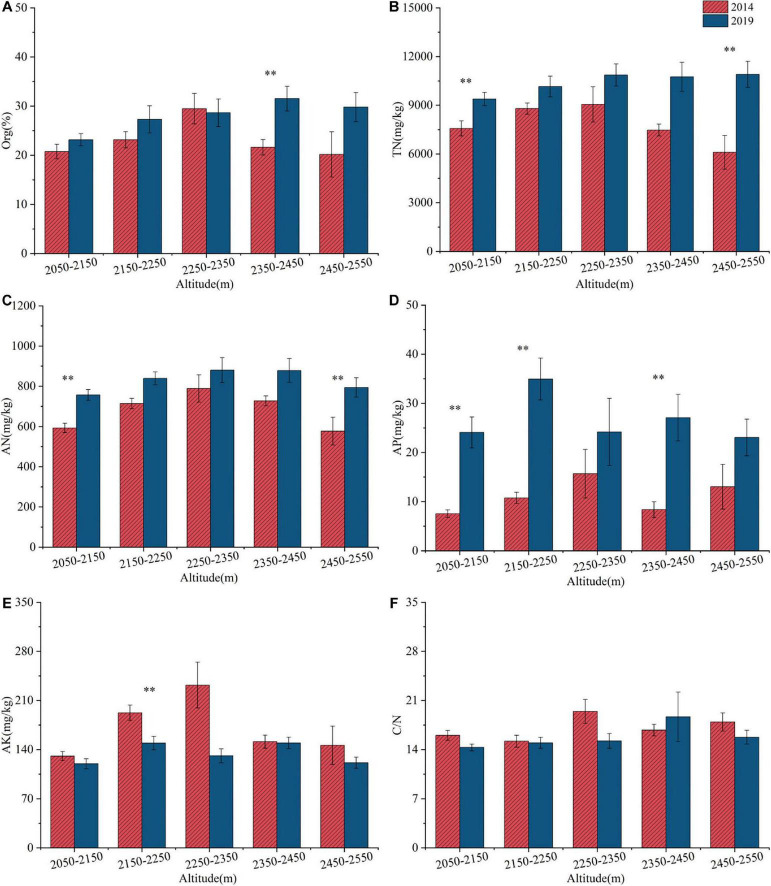
Changes in soil nutrient contents [Org **(A)**, TN **(B)**, AN **(C)**, AP **(D)**, AK **(E)**, C/N **(F)**] at different altitudes from 2014 to 2019 in the ATCBM. **represents a difference that is significant at the 0.05 level.

Between 2014 and 2019, the distributions of Org, TN, AN, AP and AK exhibited a pattern of initially increasing and then decreasing with increasing elevation (Figure 6). From 2014 to 2019, Org, TN, AN, and AP generally increased across elevation gradients, with the exception of Org in the 2250–2350 m range. Notably, significant increases (*p* < 0.05) were observed in the following elevation ranges: Org within 2350–2450 m; TN within 2050–2150 m and 2450–2550 m; AN within 2050–2150 m and 2450–2550 m; and AP within 2050–2150 m, 2150–2250 m, and 2450–2550 m. AK and C/N decreased across most elevation gradients. However, C/N increased within the 2350–2450 m range. Notably, AK experienced a significant decrease (*p* < 0.05) in the 2150–2250 m range.

Soil nutrient content showed different changes across various plant communities from 2014 to 2019 (Table 2). Specifically, the *D. angustifolia* community exhibited significant increases (*p* < 0.05) in TN, AN, and AP. In contrast, the *S. sitchensis* community experienced a significant decline (*p* < 0.05) in AK. The *R. chrysanthum* community displayed significant increases (*p* < 0.05) in TN, AN, and AP, along with a significant decrease (*p* < 0.05) in AK. Significant increases (*p* < 0.05) were observed in Org, TN, AN, and AP within the *V. uliginosum* community. Finally, in the *D. octopetala* community, Org, TN, AN, and AP all showed significant increases (*p* < 0.05), whereas C/N significantly decreased (*p* < 0.05).

**TABLE 2:** Changes in soil nutrient content (Org, TN, AN, AP, AK, and C/N) in typical plant communities in the ATCBM.

Plant communities	Org	TN	AN	AP	AK	C/N
*D. angustifolia*	4.22 ± 2.12	2554.76 ± 933.92**	223.68 ± 93.92**	14.37 ± 5.16**	5.38 ± 14.47	−1.28 ± 1.08
*S. sitchensis*	0.55 ± 1.77	870.55 ± 602.67	57.33 ± 29.73	8.02 ± 4.18	−70.12 ± 12.74**	−1.28 ± 0.78
*R. chrysanthum*	4.88 ± 1.76**	2033.64 ± 500.86**	167.11 ± 9.65**	24.51 ± 2.86**	−26.08 ± 11.99**	−0.52 ± 1.03
*V. uliginosum*	10.14 ± 3.92**	4263.87 ± 723.02**	205.33 ± 55.41**	17.17 ± 4.71**	−8.84 ± 14.55	−1.57 ± 1.76
*D. octopetala*	7.33 ± 2.96*	4203.83 ± 970.87**	171.17 ± 58.66*	12.18 ± 4.98*	−24.15 ± 25.35	−2.34 ± 0.67**

**Represents a difference that is significant at the 0.05 level.

### 3.3 Driving forces of tundra vegetation changes

**FIGURE 7:**
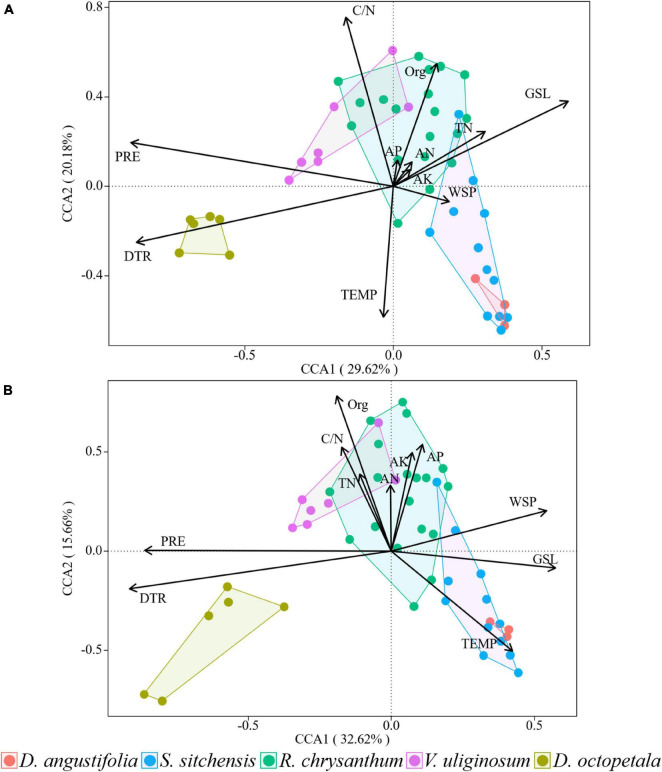
Canonical correspondence analysis of the distributions of plant communities and environmental factors in 2014 **(A)** and 2019 **(B)** over the ATCBM.

Through canonical correspondence analysis, we observed that the explanatory power of environmental factors on plant community distribution remained consistent across both surveys, with the dominant factors largely unchanged. In 2014, axes 1 and 2 together explained 49.8% of the variation in plant community distribution (Figure 7A), while in 2019, they explained 48.28% of the variation (Figure 7B). The environmental factors most influential on plant community distribution in 2014 were DTR, PRE, GSL, and C/N. By 2019, the dominant factors shifted slightly to include DTR, TEMP, Org, and PRE. We could find that climate factors, rather than soil nutrients, dominated the distribution of plant communities.

**FIGURE 8:**
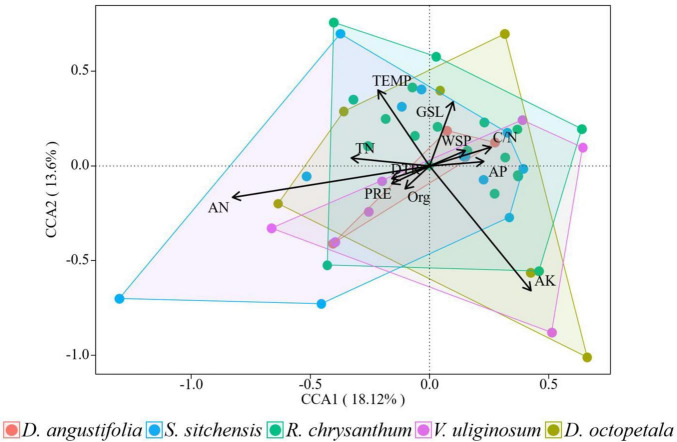
Canonical correspondence analysis of the changes of plant communities and environmental factors from 2014 to 2019 over the ATCBM.

Compared to their explanatory power for plant community distribution, environmental factors demonstrated a lower explanatory power for changes in plant community from 2014 to 2019. Axes 1 and 2 together explained 31.72% of these changes in plant communities (Figure 8). The AN factor, indicated by the longest arrow in the analysis, had the greatest impact on these changes. Among the 11 environmental factors assessed, the impact on changes in the five plant communities, ranked from largest to smallest, were as follows: AK, AN, TEMP, GSL, TN, C/N, PRE, AP, WSP, DTR, and Org. Soil nutrients exhibited a greater impact than climate factors on the short-term changes in plant community.

## 4 Discussion

### 4.1 Changes in the importance value of plant species

Permanent plot surveys offer a nuanced examination of shifts in the composition and structure of tundra vegetation, providing a detailed contrast to remote sensing methods, which generally capture only large-scale greening or browning trends in ecosystems with lower precision (Myers-Smith et al., 2019). We observed a significant decrease in the IVI of typical dwarf shrub species in the alpine tundra, including *R. chrysanthum*, *V. uliginosum*, and *D. octopetala*. Conversely, the IVI of herbaceous plants such as *D. angustifolia* and *S. sitchensis* exhibited noticeable increases. This change was primarily attributed to the gradual encroachment of herbaceous plants, such as *D. angustifolia* and *S. sitchensis*, into the traditional growth areas of tundra vegetation (Jin et al., 2019a). This indicated intense competition between the native dwarf shrub species of the tundra and encroaching herbaceous species. It confirmed that the composition and structure of tundra vegetation had undergone significant changes in a short period under the pressure of environmental changes, with a trend towards meadowization (Wang et al., 2019). Moreover, this change further led to the fragmentation of the alpine tundra landscape, contrasting with the trend of tundra shrubification observed in polar and high-latitude regions in the past (Sturm et al., 2001; Cannone et al., 2007; Formica et al., 2014; Schore et al., 2023).

### 4.2 Changes in plant species diversity along elevation gradients

This study observed a decrease in plant species richness with increasing altitude, consistent with findings from other research (Kazakis et al., 2006; Vanneste et al., 2017; Hamid et al., 2020). This was attributed to temperature directly influencing the ecological niches of plants, affecting their distribution and diversity (Waldock et al., 2018). Additionally, as the terrain’s slope increased with altitude, the steeper areas became more susceptible to erosion, thereby reducing the potential growth areas for vegetation (Theurillat et al., 2007). Species diversity and evenness peaked at elevations between 2250 and 2350 m, coinciding with the upper boundary expansion of the encroaching herbaceous species. Although there were signs of encroachment from lower-altitude plant species within this range, the native tundra plat species still dominated. In this region, although the number of species was not the highest, each species had relatively high abundance, and species distribution was relatively even (Han et al., 2023). Consistent with our hypothesis, species diversity increased over time (Steinbauer et al., 2018; Salminen et al., 2023; Zemlianskii et al., 2024), but the degree of change varied across different altitude gradients. Over a five-year period, the greatest changes in species richness and diversity were observed between 2150 m and 2250 m, where six plant species were added and the diversity index increased by 0.36. This was due to the area serving as a mixing zone, where encroaching plant species and native tundra plant species intermingled. Many plants migrated upwards from lower altitudes, intensifying resource competition between encroaching and native species, which ultimately resulted in significant changes in species composition (Dolezal et al., 2016).

### 4.3 Changes in species composition and coverage of typical plant communities

Plant encroachments significantly influenced the composition and dynamics of communities (Fahey et al., 2020; Livingstone et al., 2020). After encroaching on the alpine tundra, *D. angustifolia* competed with the native dwarf shrub communities, leading to significant changes in the composition and structure of the native tundra plant communities. Species richness increased in these communities of *R. chrysanthum*, *V. uliginosum*, and *D. octopetala*, while it decreased in the communities of *D. angustifolia* and *S. sitchensis*. This may confirm that environment change led to the homogenization of previously heterogeneous tundra plant communities (Stewart et al., 2018). The coverages of dominant species were decreasing to different degrees in the other four plant communities, except for an increase the *D. angustifolia* community. Moreover, the coverage of *D. angustifolia* increased within the *S. sitchensis* and *R. chrysanthum* communities, while *S. sitchensis* coverage also expanded within the *D. angustifolia* and *R. chrysanthum* communities. This indicated that both low-altitude herbaceous plants, such as *D. angustifolia*, and native tundra herbaceous species like *S. sitchensis*, were expanding in the alpine tundra. This expansion contributed to a shift in plant community composition toward herbaceous dominance, ultimately leading to the meadowization of the tundra. In this competitive process, low the dwarf shrubs like *R. chrysanthum* and *V. uliginosum* in lower altitude were at risk of disappearing (Jin et al., 2015; Zong et al., 2016; Wang et al., 2019).

### 4.4 Analysis of drivers of tundra vegetation change

Vegetation changes were closely related to environmental changes (Dolezal et al., 2016). With climate-induced changes in abiotic mountain environmental factors, the structure and distribution of mountain plants also changed accordingly (López-Angulo et al., 2019; Zu and Wang, 2022). Numerous studies have attributed the distribution of alpine tundra plant communities primarily to global warming (Lenoir and Svenning, 2014), which was confirmed in this study. Both surveys confirmed that climate factors such as PRE, DTR, and TEMP were primary drivers of plant community distribution, surpassing the influence of soil nutrients. However, our findings indicated although climate factors showed some influence on vegetation dynamics, the principal drivers of changes in plant communities during the study period were not climate factors but rather shifts in soil nutrients, particularly AN and AK. Increasing nitrogen levels in alpine tundra areas has been observed to promote the growth of herbaceous plants, while simultaneously leading to a reduction in the population of local dwarf shrubs (Nilsson et al., 2002). Zong et al. (2016) also found that nutrient perturbation had a greater impact than temperature on the expansion of *D. angustifolia* in the ATCBM, which was consistent with our results.

### 4.5 Potential mechanisms of soil nutrient changes mediated by microbial communities

**FIGURE 9:**
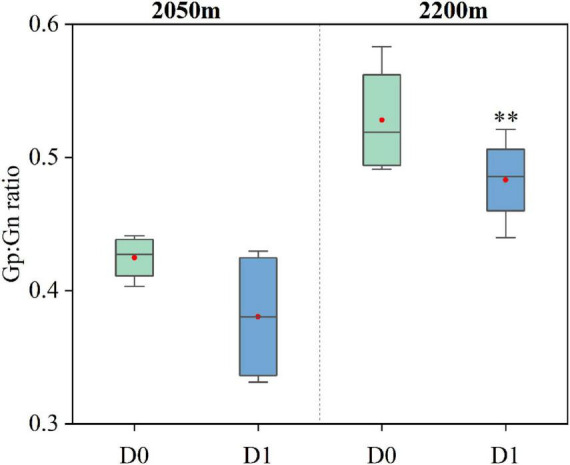
Changes in the ratio of Gram-positive to Gram-negative bacteria in soil microbial communities at different elevations in the ATCBM between areas with and without *D. angustifolia* encroachment (D0 = no *D. angustifolia* encroachment, D1 = *D. angustifolia* encroachment). **represents a difference that is significant at the 0.05 level.

Changes in soil nutrients were influenced by shifts in the soil microbial communities (Eskelinen et al., 2009; Philippot et al., 2024). Although the temperature change was not significant during the study period, the relatively higher temperatures resulting from decades of warming were stimulating microbial decomposition activity in the ATCBM (Jin et al., 2019b). This heightened microbial activity could accelerate the breakdown of organic matter, thereby affecting soil nutrient levels (Maes et al., 2024). Additionally, the trend towards meadowization of the alpine tundra intensified with the encroachment of *D. angustifolia*, resulting in an increase in herbaceous vegetation. Herbaceous plants contained higher proportions of easily degradable compounds such as monosaccharides and proteins, making their litter more prone to decomposition (Hobbie, 1996; Dorrepaal et al., 2005). At different elevations in our study area, the ratio of Gram-positive to Gram-negative bacteria in the soil microbial communities was significantly lower in areas with *D. angustifolia* encroachment compared to areas without such encroachment (Figure 9). The proportion of Gram-positive bacteria specialized in decomposing recalcitrant substrates, such as the litter of *R. chrysanthum* rich in lignin, cellulose, and other recalcitrant organic compounds, decreased in the microbial community, while the proportion of Gram-negative bacteria specialized in decomposing easily degradable substrates increased (Li et al., 2023). Such microbial changes accelerated the decomposition of herbaceous plant substrates, leading to an increase in nutrient cycling rates, a decrease in soil C/N, and an increase in available nutrients (Li et al., 2017). These changes in soil nutrients further affected vegetation changes (Zhang et al., 2018), promoting the further expansion of herbaceous plants in the alpine tundra. However, due to experimental limitations, this study was unable to separately investigate how changes in soil microorganisms affected changes in soil physicochemical properties.

### 4.6 Study implications and limitations

In this study, detailed monitoring of permanent plots in the ATCBM revealed significant short-term changes in tundra plant communities. Soil nutrient changes, rather than climate change, were identified as the main drivers of these vegetation changes. This is particularly notable given that the ATCBM is located in the core area of a national nature reserve, unaffected by agricultural or grazing activities, highlighting the importance of natural factors in influencing alpine tundra vegetation dynamics. Our regular plot surveys effectively pinpointed the actual drivers of vegetation changes, addressing the limitations inherent in controlled experiments (Clark et al., 2007). However, our study faced certain limitations and uncertainties. Although the three plot replicates at each altitude range were spaced only 25 m apart, the variations in elevation, slope, and vegetation types across different sites ensured the independence of the data. Future studies would benefit from expanding the study area and increasing the number of sample plots to enhance data representativeness and reliability. Additionally, due to the complexity of the alpine environment, we resorted to using interpolation methods to acquire precipitation data. Although these interpolation results were highly correlated with observed data, they still had some uncertainties, necessitating more precise climate data for future analyses (Tapiador et al., 2017). Continuous monitoring of tundra vegetation changes will enable managers to more effectively identify and address ecological challenges, devising robust strategies to respond to future environmental shifts (Reid et al., 2022). This proactive approach is crucial for maintaining the health and stability of the tundra ecosystem, thereby ensuring its long-term sustainability (Rani et al., 2020).

## 5 Conclusion

This study investigated vegetation changes and their dominant drivers in the ATCBM over a short period by re-surveying permanent plots in 2019 and comparing them with data from 2014. The results revealed significant changes in alpine tundra vegetation during the study period. The importance values of typical alpine tundra plants such as *R. chrysanthum*, *V. uliginosum*, and *D. octopetala* decreased noticeably, while those of herbaceous species such as *D. angustifolia* and *S. sitchensis* increased significantly. Species richness, diversity, and evenness at different altitudinal gradients showed varying degrees of increase. A distinct expansion trend of herbaceous species was observed in the alpine tundra, contributing to a shift in plant community composition toward herbaceous dominance. This shift might result in the meadowization of the dwarf shrub tundra. Soil nutrients rather than climate factors, dominated the changes of plant communities over a short period. These findings provide scientific references for the conservation and management of biodiversity, as well as for projecting future vegetation dynamics in alpine tundra. Furthermore, to accurately simulate vegetation changes and reduce uncertainties in alpine tundra, our findings suggest that vegetation dynamic models should take into account the effect of soil nutrient variation on short-term changes in plant communities.

## Data Availability

The raw data supporting the conclusions of this article will be made available by the authors, without undue reservation.

## References

[B1] Alatalo J. M. Jagerbrand A. K. Juhanson J. Michelsen A. Luptacik P. (2017). Impacts of twenty years of experimental warming on soil carbon, nitrogen, moisture and soil mites across alpine/subarctic tundra communities. *Sci. Rep.* 7:44489. 10.1038/srep44489 28295022 PMC5353735

[B2] Bligh E. G. Dyer W. J. (1959). A rapid method of total lipid extraction and purification. *Can. J. Biochem. Physiol.* 37 911–917. 10.1139/o59-099 13671378

[B3] Bremner J. M. Mulvaney C. (1982). “Nitrogen—total,” in *Methods of soil analysis. Part 2. Chemical and microbiological properties*, Vol. 9 eds Page A. L. Miller R. H. Keeney D. R. (Madison, WI: American Society of Agronomy, Soil Science Society of America), 595–624.

[B4] Britton A. J. Fisher J. M. (2008). Growth responses of low-alpine dwarf-shrub heath species to nitrogen deposition and management. *Environ. Pollut.* 153 564–573. 10.1016/j.envpol.2007.09.022 17988771

[B5] Cannone N. Sgorbati S. Guglielmin M. (2007). Unexpected impacts of climate change on alpine vegetation. *Front. Ecol. Environ.* 5:360–364. 10.1890/1540-929520075[360:Uiocco]2.0.Co;2

[B6] Chu C. Kleinhesselink A. R. Havstad K. M. McClaran M. P. Peters D. P. Vermeire L. T. (2016). Direct effects dominate responses to climate perturbations in grassland plant communities. *Nat. Commun.* 7:11766. 10.1038/ncomms11766 27273085 PMC4899860

[B7] Clark C. M. Cleland E. E. Collins S. L. Fargione J. E. Gough L. Gross K. L. (2007). Environmental and plant community determinants of species loss following nitrogen enrichment. *Ecol. Lett.* 10 596–607. 10.1111/j.1461-0248.2007.01053.x 17542938

[B8] Czortek P. Eycott A. E. Grytnes J.-A. Delimat A. Kapfer J. Jaroszewicz B. (2018). Effects of grazing abandonment and climate change on mountain summits flora: A case study in the Tatra Mts. *Plant Ecol.* 219 261–276. 10.1007/s11258-018-0794-6

[B9] Danby R. K. Koh S. Hik D. S. Price L. W. (2011). Four decades of plant community change in the Alpine tundra of southwest Yukon, Canada. *Ambio* 40 660–671. 10.1007/s13280-011-0172-2 21954728 PMC3357857

[B10] Dolezal J. Dvorsky M. Kopecky M. Liancourt P. Hiiesalu I. Macek M. (2016). Vegetation dynamics at the upper elevational limit of vascular plants in Himalaya. *Sci. Rep.* 6:24881. 10.1038/srep24881 27143226 PMC4855180

[B11] Dorrepaal E. Cornelissen J. H. C. Aerts R. WallÉN B. O. Van Logtestijn R. S. P. (2005). Are growth forms consistent predictors of leaf litter quality and decomposability across peatlands along a latitudinal gradient? *J. Ecol.* 93 817–828. 10.1111/j.1365-2745.2005.01024.x

[B12] Du H. Liu J. Li M. H. Büntgen U. Yang Y. Wang L. (2017). Warming-induced upward migration of the alpine treeline in the Changbai Mountains, northeast China. *Glob. Change Biol.* 24 1256–1266. 10.1111/gcb.13963 29080270

[B13] Dunwiddie P. W. J. A. Research A. (1977). Recent tree invasion of subalpine meadows in the Wind River Mountains. *Wyoming.* 9 393–399. 10.1080/00040851.1977.12003932

[B14] Epstein H. E. Raynolds M. K. Walker D. A. Bhatt U. S. Tucker C. J. Pinzon J. E. (2012). Dynamics of aboveground phytomass of the circumpolar Arctic tundra during the past three decades. *Environ. Res. Lett.* 7:5506. 10.1088/1748-9326/7/1/015506

[B15] Eskelinen A. Stark S. Mannisto M. (2009). Links between plant community composition, soil organic matter quality and microbial communities in contrasting tundra habitats. *Oecologia* 161 113–123. 10.1007/s00442-009-1362-5 19452173

[B16] Fahey C. Koyama A. Antunes P. M. Dunfield K. Flory S. L. (2020). Plant communities mediate the interactive effects of invasion and drought on soil microbial communities. *ISME J.* 14 1396–1409. 10.1038/s41396-020-0614-6 32076127 PMC7242364

[B17] Formica A. Farrer E. C. Ashton I. W. Suding K. N. (2014). Shrub Expansion over the past 62 years in rocky mountain alpine tundra: Possible causes and consequences. *Arctic Antarctic Alpine Res.* 46 616–631. 10.1657/1938-4246-46.3.616 38960936

[B18] Frostegård Å Tunlid A. Bååth E. (1991). Microbial biomass measured as total lipid phosphate in soils of different organic content. *J. Microbiol. Methods* 14 151–163. 10.1016/0167-7012(91)90018-l

[B19] Gazol A. Moiseev P. Camarero J. J. (2017). Changes in plant taxonomic and functional diversity patterns following treeline advances in the South Urals. *Plant Ecol. Divers.* 10 283–292. 10.1080/17550874.2017.1400126

[B20] Grabherr G. Gottfried M. Pauli H. (2010). Climate change impacts in alpine environments. *Geogr. Compass* 4 1133–1153. 10.1111/j.1749-8198.2010.00356.x

[B21] Greig-Smith P. (1983). *Quantitative plant ecology.* Berkeley, CA: University of California Press.

[B22] Hallinger M. Manthey M. Wilmking M. (2010). Establishing a missing link: Warm summers and winter snow cover promote shrub expansion into alpine tundra in Scandinavia. *New Phytol.* 186 890–899. 10.1111/j.1469-8137.2010.03223.x 20345642

[B23] Hamid M. Khuroo A. A. Malik A. H. Ahmad R. Singh C. P. Dolezal J. (2020). Early evidence of shifts in alpine summit vegetation: A case study from Kashmir Himalaya. *Front. Plant Sci.* 11:421. 10.3389/fpls.2020.00421 32391033 PMC7194130

[B24] Han J. Yin H. Xue J. Zhang Z. Xing Z. Wang S. (2023). Vertical distribution differences of the understory herbs and their driving factors on shady and sunny slopes in high altitude mountainous areas. *Front. Forests Glob. Change* 6:8317. 10.3389/ffgc.2023.1138317

[B25] Hao Z. Dai Limin H. S. H. David J. M. Guofan S. J. (2001). Potential response of major tree species to climate warming in Changbai Mountain, Northeast China. *Chin. J. Appl. Ecol.* 12:653.

[B26] Hobbie S. E. (1996). temperature and plant species control over litter decomposition in Alaskan Tundra. *Ecol. Monogr.* 66 503–522. 10.2307/2963492

[B27] Holzinger B. Hülber K. Camenisch M. Grabherr G. (2007). Changes in plant species richness over the last century in the eastern Swiss Alps: Elevational gradient, bedrock effects and migration rates. *Plant Ecol.* 195 179–196. 10.1007/s11258-007-9314-9

[B28] Huang X.-C. Li C.-H. (1984). An analysis on the ecology of alpine tundra landscape of Changbai Mountains. *Acta Geogr. Sin. Beijing* 39 285–297.

[B29] Jin Y. Xu J. He H. Li M. H. Tao Y. Zhang Y. (2019a). The Changbai alpine shrub tundra will be replaced by herbaceous tundra under global climate change. *Plants (Basel)* 8 370. 10.3390/plants8100370 31557891 PMC6843343

[B30] Jin Y. Zhang Y. Xu Z. Gu X. Xu J. Tao Y. (2019b). Soil microbial community and enzyme activity responses to herbaceous plant expansion in the Changbai mountains Tundra, China. *Chin. Geogr. Sci.* 29 985–1000. 10.1007/s11769-019-1067-6

[B31] Jin Y. Xu J. Wang Y. Wang S. Chen Z. Huang X. (2015). Effects of nitrogen deposition on tundra vegetation undergoing invasion by *Deyeuxia angustifolia* in Changbai Mountains. *Chin. Geogr. Sci.* 26 99–108. 10.1007/s11769-015-0746-1

[B32] Ju J. Masek J. G. (2016). The vegetation greenness trend in Canada and US Alaska from 1984–2012 Landsat data. *Remote Sens. Environ.* 176 1–16. 10.1016/j.rse.2016.01.001

[B33] Kapfer J. Hedl R. Jurasinski G. Kopecky M. Schei F. H. Grytnes J. A. (2016). Resurveying historical vegetation data - opportunities and challenges. *Appl. Veg. Sci.* 20 164–171. 10.1111/avsc.12269 30245580 PMC6145442

[B34] Kazakis G. Ghosn D. Vogiatzakis I. N. Papanastasis V. P. (2006). Vascular plant diversity and climate change in the alpine zone of the Lefka Ori, Crete. *Biodivers. Conserv.* 16 1603–1615. 10.1007/s10531-006-9021-1

[B35] Klein J. A. Harte J. Zhao X. Q. (2004). Experimental warming causes large and rapid species loss, dampened by simulated grazing, on the Tibetan Plateau. *Ecol. Lett.* 7 1170–1179. 10.1111/j.1461-0248.2004.00677.x

[B36] Körner C. (1999). *Alpine plant life: Functional plant ecology of high mountain ecosystems.* Berlin: Springer, 10.1007/978-3-642-18970-8

[B37] Lagergren F. Björk R. G. Andersson C. Belušić D. Björkman M. P. Kjellström E. (2024). Kilometre-scale simulations over Fennoscandia reveal a large loss of tundra due to climate warming. *Biogeosciences* 21 1093–1116. 10.5194/bg-21-1093-2024

[B38] Lajtha K. Jarrell W. (1999). *Soil phosphorus.* Oxford: Oxford University Press, 115–142.

[B39] Lenoir J. Svenning J. C. (2014). Climate-related range shifts – a global multidimensional synthesis and new research directions. *Ecography* 38 15–28. 10.1111/ecog.00967

[B40] Li L. Xing M. Lv J. Wang X. Chen X. (2017). Response of rhizosphere soil microbial to Deyeuxia angustifolia encroaching in two different vegetation communities in alpine tundra. *Sci. Rep.* 7:43150. 10.1038/srep43150 28220873 PMC5318906

[B41] Li N. Du H. Li M.-H. Na R. Dong R. He H. S. (2023). *Deyeuxia angustifolia* upward migration and nitrogen deposition change soil microbial community structure in an alpine tundra. *Soil Biol. Biochem.* 180:109009. 10.1016/j.soilbio.2023.109009

[B42] Livingstone S. W. Isaac M. E. Cadotte M. W. (2020). Invasive dominance and resident diversity: Unpacking the impact of plant invasion on biodiversity and ecosystem function. *Ecol. Monogr.* 90:1425. 10.1002/ecm.1425

[B43] López-Angulo J. Pescador D. S. Sánchez A. M. Luzuriaga A. L. Cavieres L. A. Escudero A. (2019). Alpine vegetation dataset from three contrasting mountain ranges differing in climate and evolutionary history. *Data Brief* 27:104816. 10.1016/j.dib.2019.104816 31788524 PMC6880020

[B44] Lu A. Zhang X. Wang S. Wang M. J. (2011). Effect of disturbance on the community species diversity in Yunding Mount. subalpine meadow. *Bull. Botan. Res.* 31 73–78.

[B45] Ma K. Huang J. Yu S. Chen L. (1995). Plant community diversity in Dongling Mountain, Beijing, China. II. Species richness, evenness and species diversities. *Acta Ecol. Sin.* 15 268–277.

[B46] Maes S. L. Dietrich J. Midolo G. Schwieger S. Kummu M. Vandvik V. (2024). Environmental drivers of increased ecosystem respiration in a warming tundra. *Nature* 629 105–113. 10.1038/s41586-024-07274-7 38632407 PMC11062900

[B47] Metcalfe D. B. Hermans T. D. G. Ahlstrand J. Becker M. Berggren M. Bjork R. G. (2018). Patchy field sampling biases understanding of climate change impacts across the Arctic. *Nat. Ecol. Evol.* 2 1443–1448. 10.1038/s41559-018-0612-5 30013133

[B48] Myers-Smith I. H. Forbes B. C. Wilmking M. Hallinger M. Lantz T. Blok D. (2011). Shrub expansion in tundra ecosystems: Dynamics, impacts and research priorities. *Environ. Res. Lett.* 6:5509. 10.1088/1748-9326/6/4/045509

[B49] Myers-Smith I. H. Grabowski M. M. Thomas H. J. D. Angers-Blondin S. Daskalova G. N. Bjorkman A. D. (2019). Eighteen years of ecological monitoring reveals multiple lines of evidence for tundra vegetation change. *Ecol. Monogr.* 89 106–117. 10.1002/ecm.1351

[B50] Nill L. Grünberg I. Ullmann T. Gessner M. Boike J. Hostert P. (2022). Arctic shrub expansion revealed by Landsat-derived multitemporal vegetation cover fractions in the Western Canadian Arctic. *Remote Sens. Environ.* 281:3228.

[B51] Nilsson M. C. Wardle D. A. Zackrisson O. Jäderlund A. (2002). Effects of alleviation of ecological stresses on an alpine tundra community over an eight-year period. *Oikos* 97 3–17. 10.1034/j.1600-0706.2002.970101.x

[B52] Niu Y. J. Yang S. W. Wang G. Z. Liu L. Hua L. M. (2017). Evaluation and selection of species diversity index under grazing disturbance in alpine mea-dow. *Ying Yong Sheng Tai Xue Bao* 28 1824–1832. 10.13287/j.1001-9332.201706.026 29745144

[B53] Olorunfemi I. Fasinmirin J. Ojo A. (2016). Modeling cation exchange capacity and soil water holding capacity from basic soil properties. *Eurasian J. Soil Sci.* 5 266–274. 10.18393/ejss.2016.4.266-274

[B54] Pauli H. Gottfried M. Dullinger S. Abdaladze O. Akhalkatsi M. Benito Alonso J. L. (2012). Recent plant diversity changes on Europe’s mountain summits. *Science* 336 353–355. 10.1126/science.1219033 22517860

[B55] Pauwels J. Van Ranst E. Verloo M. Mvondo Ze A. (1992). *Méthodes d’analyses de sols et de plantes, équipement, gestion de stocks de verrerie et de produits chimiques.* Bruxelles: Publications Agricoles.

[B56] Philippot L. Chenu C. Kappler A. Rillig M. C. Fierer N. (2024). The interplay between microbial communities and soil properties. *Nat. Rev. Microbiol.* 22 226–239. 10.1038/s41579-023-00980-5 37863969

[B57] Pickering C. Hill W. Green K. (2008). Vascular plant diversity and climate change in the alpine zone of the Snowy Mountains, Australia. *Biodivers. Conserv.* 17 1627–1644. 10.1007/s10531-008-9371-y

[B58] Rani G. Kaur J. Kumar A. Yogalakshmi K. (2020). “Ecosystem health and dynamics: An indicator of global climate change,” in *Contemporary environmental issues and challenges in era of climate change*, eds Singh P. Singh R. Srivastava V. (Singapore: Springer), 1–32. 10.1007/978-981-32-9595-7_1

[B59] Rantanen M. Karpechko A. Y. Lipponen A. Nordling K. Hyvärinen O. Ruosteenoja K. (2022). The Arctic has warmed nearly four times faster than the globe since 1979. *Commun. Earth Environ.* 3:168. 10.1038/s43247-022-00498-3

[B60] Reichle L. M. Epstein H. E. Bhatt U. S. Raynolds M. K. Walker D. A. (2018). Spatial heterogeneity of the temporal dynamics of arctic tundra vegetation. *Geophys. Res. Lett.* 45 9206–9215. 10.1029/2018gl078820

[B61] Reid K. A. Reid D. G. Brown C. D. (2022). Patterns of vegetation change in Yukon: Recent findings and future research in dynamic subarctic ecosystems. *Environ. Rev.* 30 380–401. 10.1139/er-2021-0110

[B62] Rixen C. Høye T. T. Macek P. Aerts R. Alatalo J. M. Anderson J. T. (2022). Winters are changing: Snow effects on Arctic and alpine tundra ecosystems. *Arctic Sci.* 8 572–608. 10.1139/as-2020-0058

[B63] Salminen H. Tukiainen H. Alahuhta J. Hjort J. Huusko K. Grytnes J.-A. (2023). Assessing the relation between geodiversity and species richness in mountain heaths and tundra landscapes. *Landsc. Ecol.* 38 2227–2240. 10.1007/s10980-023-01702-1

[B64] Schore A. I. G. Fraterrigo J. M. Salmon V. G. Yang D. Lara M. J. (2023). Nitrogen fixing shrubs advance the pace of tall-shrub expansion in low-Arctic tundra. *Commun. Earth Environ.* 4:421. 10.1038/s43247-023-01098-5

[B65] Shevtsova I. Heim B. Kruse S. Schröder J. Troeva E. I. Pestryakova L. A. (2020). Strong shrub expansion in tundra-taiga, tree infilling in taiga and stable tundra in central Chukotka (north-eastern Siberia) between 2000 and 2017. *Environ. Res. Lett.* 15:ab9059. 10.1088/1748-9326/ab9059

[B66] Šmilauer P. Lepš J. (2014). *Multivariate analysis of ecological data using CANOCO 5.* Cambridge: Cambridge University Press.

[B67] Steinbauer M. J. Grytnes J. A. Jurasinski G. Kulonen A. Lenoir J. Pauli H. (2018). Accelerated increase in plant species richness on mountain summits is linked to warming. *Nature* 556 231–234. 10.1038/s41586-018-0005-6 29618821

[B68] Stewart L. Simonsen C. E. Svenning J. C. Schmidt N. M. Pellissier L. (2018). Forecasted homogenization of high Arctic vegetation communities under climate change. *J. Biogeogr.* 45 2576–2587. 10.1111/jbi.13434

[B69] Sturm M. Racine C. Tape K. (2001). Climate change. Increasing shrub abundance in the Arctic. *Nature* 411 546–547. 10.1038/35079180 11385559

[B70] Tape K. E. N. Sturm M. Racine C. (2006). The evidence for shrub expansion in Northern Alaska and the Pan-Arctic. *Glob. Change Biol.* 12 686–702. 10.1111/j.1365-2486.2006.01128.x

[B71] Tapiador F. J. Navarro A. Levizzani V. García-Ortega E. Huffman G. J. Kidd C. (2017). Global precipitation measurements for validating climate models. *Atmosph. Res.* 197 1–20. 10.1016/j.atmosres.2017.06.021

[B72] Tarnocai C. Canadell J. G. Schuur E. A. G. Kuhry P. Mazhitova G. Zimov S. (2009). Soil organic carbon pools in the northern circumpolar permafrost region. *Glob. Biogeochem. Cycles* 23:3327. 10.1029/2008gb003327 30683748

[B73] Theurillat J.-P. Iocchi M. Cutini M. De Marco G. (2007). Vascular plant richness along an elevation gradient at Monte Velino (Central Apennines, Italy). *Biogeographia* 28:3. 10.21426/b628110003

[B74] Vanneste T. Michelsen O. Graae B. J. Kyrkjeeide M. O. Holien H. Hassel K. (2017). Impact of climate change on alpine vegetation of mountain summits in Norway. *Ecol. Res.* 32 579–593. 10.1007/s11284-017-1472-1

[B75] Waldock C. Dornelas M. Bates A. E. (2018). Temperature-driven biodiversity change: Disentangling space and time. *Bioscience* 68 873–884. 10.1093/biosci/biy096 30464352 PMC6238962

[B76] Walker D. A. Daniëls F. J. A. Matveyeva N. V. Šibík J. Walker M. D. Breen A. L. (2018). Circumpolar arctic vegetation classification. *Phytocoenologia* 48 181–201. 10.1127/phyto/2017/0192

[B77] Walker M. D. Wahren C. H. Hollister R. D. Henry G. H. Ahlquist L. E. Alatalo J. M. (2006). Plant community responses to experimental warming across the tundra biome. *Proc. Natl. Acad. Sci. U.S.A.* 103 1342–1346. 10.1073/pnas.0503198103 16428292 PMC1360515

[B78] Walkley A. Black I. A. (1934). An examination of the Degtjareff method for determining soil organic matter, and a proposed modification of the chromic acid titration method. *Soil Sci.* 37 29–38.

[B79] Walther G. R. Beißner S. Burga C. A. (2005). Trends in the upward shift of alpine plants. *J. Veg. Sci.* 16 541–548. 10.1111/j.1654-1103.2005.tb02394.x

[B80] Wang L. Wang W. J. Wu Z. Du H. Zong S. Ma S. (2019). Potential distribution shifts of plant species under climate change in Changbai Mountains, China. *Forests* 10:498. 10.3390/f10060498

[B81] Zemlianskii V. Brun P. Zimmermann N. E. Ermokhina K. Khitun O. Koroleva N. (2024). Current and past climate co-shape community-level plant species richness in the Western Siberian Arctic. *Ecol. Evol.* 14:e11140. 10.1002/ece3.11140 38495434 PMC10944673

[B82] Zhang L. Jing Y. Xiang Y. Zhang R. Lu H. (2018). Responses of soil microbial community structure changes and activities to biochar addition: A meta-analysis. *Sci. Total Environ.* 643 926–935. 10.1016/j.scitotenv.2018.06.231 29960229

[B83] Zong S. Jin Y. Xu J. Wu Z. He H. Du H. (2016). Nitrogen deposition but not climate warming promotes *Deyeuxia angustifolia* encroachment in alpine tundra of the Changbai Mountains, Northeast China. *Sci. Total Environ.* 544 85–93. 10.1016/j.scitotenv.2015.11.144 26657251

[B84] Zu K. Wang Z. (2022). Research progress on the elevational distribution of mountain species in response to climate change. *Biodivers. Sci.* 30:21451. 10.17520/biods.2021451 34063014

